# 

**Published:** 1919-11-01

**Authors:** 


### Recent Official Publications.

(To be obtained from H.M. Stationery Office, Imperial House, Kingsway, W.C. 2. Prices include Postage.)

Ministry of Health. The Training of Health Visitors, especially for Maternity and Child Welfare Work. 1½d.
The Constitution of the Central Midwives Board. 1½d.

Tuberculosis (Sanatoria for Soldiers) Report of the Inter-Departmental Committee. 8d.

Venereal Diseases: Note by the Chairman of the Inter-Departmental Committee on Infectious Diseases in connection with Demobilisation, on Prophylaxis against Venereal Disease. 4½d.

Ministry of Pensions. Suggestions for the Care of Malaria Patients. 2d.

### PASSING EVENTS AND TOPICS.

### A "Boxing" Cot.

AN attempt is being made by the railway employees at Acton to endow a cot in the local hospital by means of a series of boxing-matches.

### A New Hospital for Officers.

"SWAYLANDS," a beautiful house in Kent, has been recently purchased by Sir Ernest Cassel, and will, after the necessary alterations have been made, be used as a hospital for officers suffering from shell-shock and nerve affections.

### Wills of Medical Men.

DR. JAMES HINSHELWOOD, surgeon to the Glasgow Eye Infirmary, and formerly assistant to the late Sir Thomas Anderson, Regius Professor of Medicine at Glasgow University, has left personal estate amounting to £16,358.

SIR BARCLAY JOSIAH BARON, M.B., C.M., of Clifton, Bristol, former Lord Mayor of Bristol, specialist on diseases of the throat, nose, and ear, a former President of the Bath and Bristol branch of the British Medical Association, and consulting physician to the Bristol General Hospital, has left £32,415.

### Hospital and Institutional Results.

**London Fever Hospital.**—Necessarily the hospital has to keep up a large establishment, whether it is needed or not. The number of available beds is 160, and the average number occupied daily last year was 42.84; the average weekly cost of each was £5 10s., as against £4 2s. 6d. in 1917.

**Edinburgh Royal Infirmary.**—The Infirmary still ends its financial year on September 30. No fewer than thirty-six legacies were received last year, amounting in the aggregate to £34,580. The ordinary income fell short of the ordinary expenditure by nearly £23,000.

**Royal Midland Institution for the Blind, Nottingham.**—The report is abbreviated for economical reasons. In respect of both the charitable and trading accounts there was a surplus in 1918. Nineteen of the twenty blind persons who left the institution during 1918 are now leading lives of useful industry.

**Royal National Hospital for Consumption, Ventnor.**—The report is printed in full and has many capital illustrations. Although the work costs £5,000 a year more than before the war, the income was sufficient last year, and allowed for some capital expenditure.

**Devonshire Hospital, Buxton.**—A businesslike note in the Committee's report calls attention to the fact that the average cost of each patient now amounts to £4 7s. 7¼d., or more than four times as much as the minimum subscription, and suggests that any subscriber under four or five guineas who sends a patient should supplement the subscription by a donation to help make up the large deficiency.

**Royal Alexandra Infirmary, Paisley.**—The directorate are of opinion that their body should not be confined to one sex, and they will gladly welcome ladies on the board.

### The Book Market.

**List of New Books Received from the following publishers:—**

Hodder and Stoughton (University of London Press): "Mental Diseases." By R. H. Cole, M.D. 15s. net.

Macmillan and Co.: "Elements of Pediatrics for Medical Students." By R. G. Freeman, A.B., M.D. 10s. 6d. net. "The Prolongation of Life." By Sir Hermann Weber, M.D., F.R.C.P. 12s. net.

A. Maloine et Fils, Paris: "Le Lait Condensé." By Dr. P. Lassoblière.

John Bale, Son and Danielsson: "The Urethroscope in the Diagnosis and Treatment of Urethritis." By Major N. P. L. Lumb, O.B.E. 10s. 6d. net.

Oxford University Press: "Menders of the Maimed." By Arthur Keith. 16s. net.

### MATRONS' AND SISTERS' APPOINTMENTS.

BECKETT HOSPITAL, BARNSLEY, YORKS.—Miss P. M. Cartmell has been appointed sister. She was trained at the Western Infirmary, Glasgow, and has recently been working under the French Flag Nursing Corps at Caen, France.

DERBYSHIRE HOSPITAL FOR SICK CHILDREN, DERBY.—Miss A. Dight has been appointed matron. She was trained at the Derbyshire Royal Infirmary, where she was afterwards sister, and she has been home sister and assistant matron at the Victoria Hospital for Children, Chelsea.

DISTRICT INFIRMARY, ASHTON-UNDER-LYNE.—Miss M. Bridge has been appointed matron. She was trained at the Derbyshire Royal Infirmary, and is at present matron of the Royal Albert Hospital, Devonport.

EXHALL TEMPORARY SANATORIUM, NEAR COVENTRY.—Miss Jean C. Alcock and Miss Margaret Murray have been appointed matron and sister respectively. Miss Alcock was trained at the Crumpsall Infirmary, Manchester, and has held appointments as staff nurse, Baguley Sanatorium, Manchester; sister and sister-in-charge, Kimlesworth Sanatorium, Yorks; and matron, Tuberculosis Dispensary, Huckley, and Mowsley Sanatorium Leicestershire. Miss Murray was trained at the Sunderland Eye Infirmary and the Blackburn Fever Hospital, and has since been sister and night sister, Penmore Hospital, Chesterfield; sister and deputy-matron at the Rothwell Isolation Hospital and Sanatorium, near Leeds.

KELLING OPEN-AIR SANATORIUM, HOLT, NORFOLK.—Miss N. Offord has been appointed sister. She was trained at St. Mary's Hospital, Paddington, and the London Homœopathic Hospital, and has been sister at the Northamptonshire Sanatorium, Creaton. Miss Offord is a member of the College of Nursing.

MUNICIPAL MATERNITY HOSPITAL, PORTSMOUTH.—Miss Edith Salisbury and Miss Estella Urry have been appointed sisters. Miss Salisbury was trained at Kettering and District General Hospital, and has since been sister at King Edward VII. Hospital, Cardiff, the British Farmers' Hospital, Brussels, and at the Auxiliary Military Hospital, Llanelly. Miss Urry was trained at the City of Westminster Infirmary, has been ward sister at Queen Charlotte's Hospital, and at the Cancer Hospital, London.

METROPOLITAN EAR, NOSE, AND THROAT HOSPITAL, FITZROY SQUARE, W. 1.—Miss M. E. Palmer has been appointed matron. She was trained at the Chelmsford and Essex Hospital, and has since been sister at the District Hospital, Watford, sister and matron of the London Throat Hospital, Great Portland Street, W.

PENMORE HOSPITAL, CHESTERFIELD.—Miss W. Drummond has been appointed sister. She was trained at the London Hospital, and has been a sister in Q.A.R.N.V.R.

TAMWORTH HOSPITAL.—Miss Lillian Reed has been appointed matron. She was trained at the Bradford Royal Infirmary, and has recently been matron of the Easton Hospital, Yorkshire.

THE INFIRMARY, BECKETT STREET, LEEDS.—Miss Elsie Fletcher has been appointed second assistant matron and home sister. She was trained at the Mill Road Infirmary, Liverpool, and has since been sister, City of Westminster Infirmary, London; sister and night sister, Maternity Hospital, Birmingham; night superintendent, City Infectious Hospital, Newcastle-on-Tyne; home sister, Townley's Military Hospital, Bolton.

UNION INFIRMARY, ROMFORD.—Miss Daisy Wilbourn and Miss Helen Ann Latham have been appointed sister-matron and deputy sister-matron respectively. Miss Wilbourn was trained at Edmonton Union Infirmary, later being charge nurse there, she has served in the Q.A.I.M.N.S.R., and has done private nursing in England and on the Continent. Miss Latham was trained at Hereford General Hospital, has been sister-in-charge at the Treloar Hospital, Alton, Hants, night superintendent at Mile End Military Hospital, and ward, maternity, theatre, night, and home sister at Shoreditch Infirmary.

UXBRIDGE UNION INFIRMARY, HILLINGDON.—Miss Rebecca J. Stevenson has been appointed night sister. She was trained at Wandsworth Infirmary, and has been maternity sister at Brentford Infirmary. Miss Stevenson has also done private nursing.

### THE COLLEGE OF NURSING (Limited by Guarantee).

### LONDON CENTRE.

### MEETING AT GUY'S HOSPITAL.

THE London Centre of the College of Nursing held a meeting at Guy's Hospital on Thursday, October 23, at 8 P.M., to review the position and define future policy. Miss Amy Hughes took the chair, and business was discussed; between forty and fifty members were present. Miss Cox-Davies, R.R.C., speaking upon the future policy, brought forward the proposal to create a Chair of Nursing and invest it with a Degree. Finances did not at present permit of its materialisation or endowment, but unauthoritatively she could promise that substantial assistance would be forthcoming to make the possibility a certainty in the future. A discussion followed upon the reasons for the entertainment, expenditure being so heavy; the best methods of raising money; and the projected meeting to form a Trades Union for Nurses. This latter suggestion was rejected without sympathy. Those present were, after the meeting, entertained to light refreshments by Sir Cooper and Lady Perry.

### SYLLABUS OF LECTURES (WINTER SESSION).

*First Course.*

THURSDAY, OCTOBER 30, "The Relation of Production to Consumption"; THURSDAY, NOVEMBER 13, "Factors in Determining Prices"; THURSDAY, DECEMBER 4, "The Problem of Unemployment." To be given by Miss Christie, London School of Economics.

*Second Course.*

THURSDAY, JANUARY 8, 1920, Public Speaking; THURSDAY, FEBRUARY 12, Public Speaking; THURSDAY, MARCH 11, a Debate (subject to be chosen by the audience on January 8) by members of the Centre, and criticised by the lecturer. To be given by Miss Bagley (head of the Polytechnic School of Speech Training).

All the above lectures will be given at the Rooms of the Medical Society, Chandos Street, Cavendish Square, at 8 P.M.

Members of the London Centre admitted on production of membership card. Non-members admitted upon payment of 6d. at the door.

---

MESSRS. EVANS SONS LESCHER AND WEBB, LTD.'S issue of ordinary shares has been substantially over-subscribed.

---

THE price of the hot-water bottle advertised in our issue of October 25 by Messrs. Henry Lewis, 2 Westmoreland Street, New Cavendish Street, W. 1, was stated to be 8s. 6d., whereas Messrs. Lewis are selling this bottle (size 12 in. by 8 in.) at 5s. 6d. each.

